# Self-expanding nitinol stents of high versus low chronic outward force in de novo femoropopliteal occlusive arterial lesions (BIOFLEX-COF trial): study protocol for a randomized controlled trial

**DOI:** 10.1186/s13063-017-2338-0

**Published:** 2017-12-14

**Authors:** Alexander Wressnegger, Alexandra Kaider, Martin A. Funovics

**Affiliations:** 1 Division of Cardiovascular and Interventional Radiology, Department of Biomedical Imaging and Image-guided Therapy, Medical University of Vienna, Wahringer Gurtel 18-20, 1090 Vienna, Austria; 2 Institute of Clinical Biometrics, Center for Medical Statistics, Informatics and Intelligent Systems, Medical University of Vienna, Wahringer Gurtel 18-20, 1090 Vienna, Austria

**Keywords:** Stent oversizing, In-stent restenosis, Neointimal hyperplasia, Chronic outward force, Femoropopliteal occlusive lesions, Peripheral arterial disease, stent

## Abstract

**Background:**

Self-expanding nitinol stents must be oversized at least by a minimal amount to ensure contact with the vessel wall and prevent migration. Once the stent is deployed it exerts a continuous force upon the vascular wall, termed chronic outward force (COF). Animal studies have found an increased neointimal hyperplasia in stents with high oversizing and thus high COF. Data about correlation between COF and neointimal hyperplasia in humans are currently lacking. The objective of the BIOFLEX-COF trial is to prospectively investigate differences in formation of intimal hyperplasia at 1 and 2 years after implantation of nitinol stents with high versus low COF in de novo femoropopliteal occlusive arterial lesions.

**Methods:**

The BIOFLEX-COF trial is a prospective, quantitative, randomized study. Eighty subjects with symptomatic peripheral arterial lesions eligible for endovascular stent implantation will be enrolled and randomly assigned to either a high COF group (LifeStent Flexstar, Bard Peripheral Vascular Inc., Tempe, AZ, USA) or low COF group (Pulsar, Biotronik AG, Bülach, Switzerland) using an online randomization program to generate a random 1:1 group allocation (block randomization). After implantation and dilatation, COF at every 2 mm along the stent axis will be calculated from the actual stent diameter versus its nominal diameter.

There will be two follow-up evaluations at 12 and 24 months. Primary endpoint is the amount of in-stent neointima at 1 year, assessed by contrast-enhanced CT angiography (CTA). In the control examinations, stent diameter and true lumen diameter will be measured on DICOM images every 2 mm along the stent axis to quantify the relative amount of in-stent restenosis.

Secondary objectives are the amount of in-stent neointima at 2 years, device- and procedure-related adverse events and target lesion revascularization (TLR) rate.

The scheduled time for recruitment is 2 years. Recruitment is expected to be complete in October 2017.

**Discussion:**

This trial is the first to prospectively investigate the influence of COF on stent patency. If successful, the results will aid in a more precise selection of stent type and size in a given target vessel.

The present study is challenging in that it compares two different self-expanding nitinol stents head-to-head against each other. To optimize the power of this study, traditional binary outcome parameters such as TLR and restenosis at Doppler ultrasound were dropped as primary endpoints. Instead, the amount of neointima inside the stent accessed by CTA was selected as (continuous) outcome parameter.

**Trial registration:**

ClinicalTrials.gov Identifier: NCT03097679. Date of registration: 14 March 2017 (retrospectively registered).

**Electronic supplementary material:**

The online version of this article (doi:10.1186/s13063-017-2338-0) contains supplementary material, which is available to authorized users.

## Background

Arterial occlusive disease in the femoropopliteal segment can be re-vascularized in an open surgical or an endovascular approach. While the surgical techniques have been optimized previously and not undergone extensive changes over the last years, endovascular techniques have been characterized by rapid revolution of a broad variety of endovascular devices that facilitate vascular access, lesion recanalization, and treatment efficacy. Simple balloon angioplasty was complemented by stent deployment and a multitude of other technologies, and reported patencies were constantly increasing. Third-generation nitinol stents show markedly improved patency compared to their early predecessors and are today used alongside stent grafts [1], drug-eluting stents and drug-coated balloons. This ever-increasing efficacy and safety of endovascular treatment in the femoropopliteal segment was reflected both in clinical practice, scientific research data, society recommendations, and patient preference by allocating ever more severe occlusive lesions to an “endo-first” strategy [2].

Unfortunately, the introduction of many new endovascular devices and techniques did not lead to a sufficient body of scientific evidence (let alone level 1 evidence) that would aid in the decision when and how to use it and which one to prefer over another. For example, indications that primary nitinol stenting is superior to balloon angioplasty and provisional stenting is based on solely three randomized control trials (RCTs) that showed superiority of primary stenting [3–5] and one RCT that showed a trend to increased patency in the stent group in short lesions [6]. The notion that drug eluting-stents might be superior to plain nitinol stents is currently based on only a single RCT in which a secondary randomization was performed between nitinol stent and drug-eluting stent after failed balloon angioplasty [7] although more studies are ongoing. Lastly, there exists not a single randomized study comparing primary nitinol stenting versus drug-covered balloon angioplasty to this day. Despite this, many researchers advocate drug-covered balloons over nitinol stents as treatment of choice in shorter lesions [8–10].

**Fig. 1:**
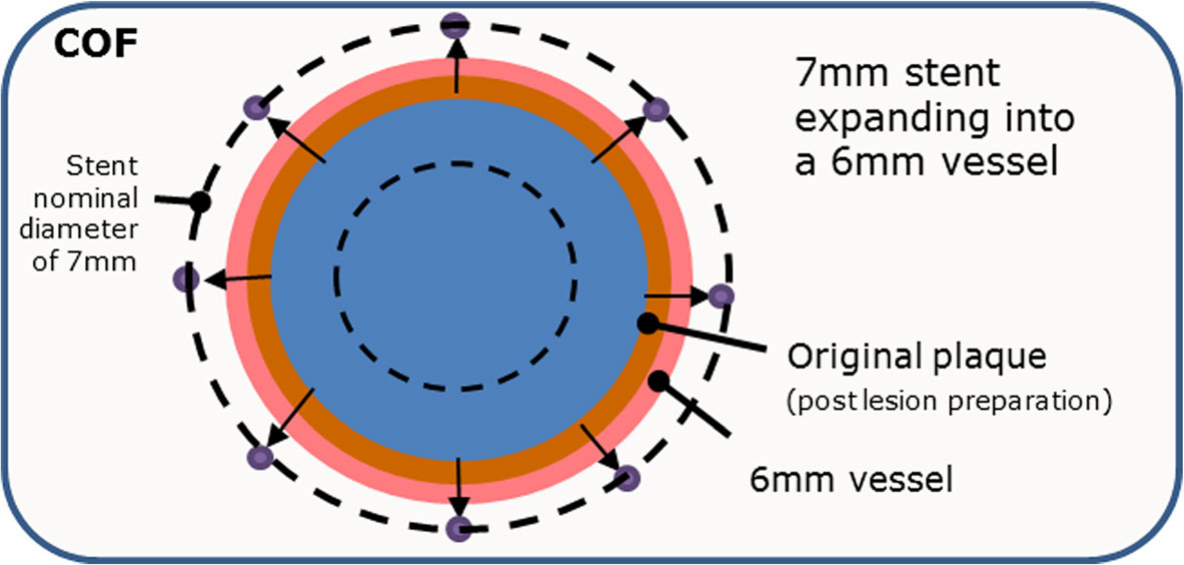
Concept of chronic outward force (COF)

The current study was planned out of a desire for more insight in how to optimally select and size a nitinol stent in the femoropopliteal segment. In contrast to drug-coated balloons or bio-absorbable devices, nitinol stents will interact with the vessel wall for the whole duration of their patency. Since nitinol stents are self-expanding, they must be oversized at least by a minimal amount to ensure contact with the vessel wall and prevent migration. Once the stent is thus deployed, the stent struts exert a continuous force upon the vascular wall, termed chronic outward force (COF) [11, 12], see Fig. 1.

**Fig. 2:**
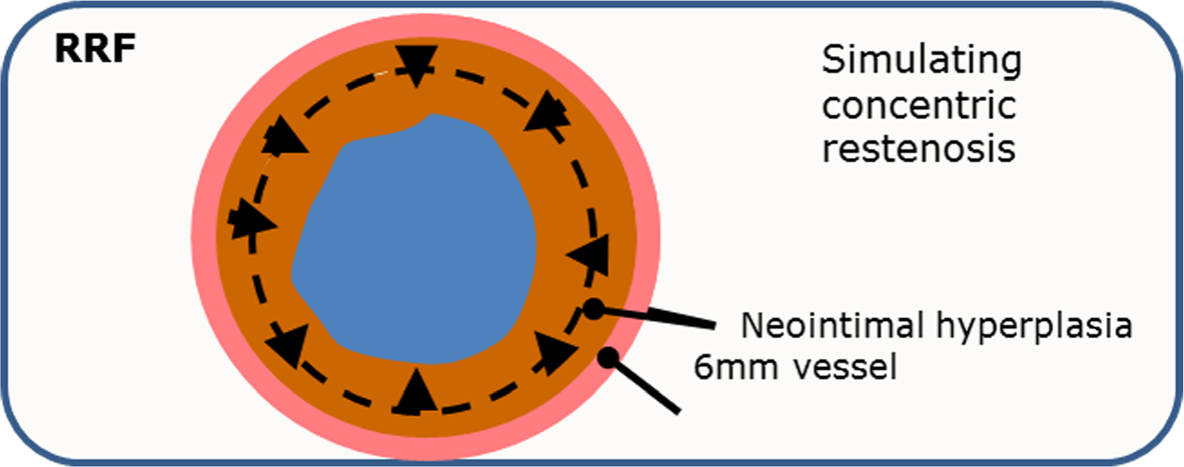
Concept of radial resistive force (RRF)

If in cases where the artery is compressed circumferentially from the outside (such as in the adductor canal) an even higher force between the stent and the vessel wall needs to be effected before the stent gives way to the external compression and is temporarily compressed. This force is called radial resistive force (RRF), see Fig. 2.

Chronic outward force depends on two factors: (i) the oversizing of the stent compared to the diameter of the target vessel (i.e., how much bigger is the nominal diameter of the stent compared to the vessel) and (ii) the spring constant (“stiffness”) of the stent material (i.e., how steep the COF increases if the stent is not allowed to expand fully to its nominal diameter) [11]. In several animal studies, groups with high versus low oversizing of self-expanding nitinol stents have been compared. The majority of the studies found a markedly increased neointimal hyperplasia in the group with high oversizing and thus high COF [13–16]. However, two studies found no significant difference between study groups [17, 18].

Data about COF and neointimal hyperplasia in humans are currently lacking. There is only one post hoc analysis of data from the VIPER study [19] that investigated patency after implantation of stent-grafts in the superficial femoral artery (SFA). At the time of the study the smallest available device was 6 mm in diameter and was implanted in vessels between 3 and 4 mm in diameter, resulting in an unusually high degree of stent oversizing. A subgroup analysis of this study collective revealed that patients with stent-grafts which had been oversized by more than 20% had 70% patency at 1 year, whereas patients with oversizing below 20% had 88% patency at 1 year (*p* = 0.047).

The objective of the BIOFLEX-COF trial is to investigate differences in formation of intimal hyperplasia at 1 and 2 years after implantation of nitinol-stents with high versus low COF in de novo femoropopliteal occlusive lesions in patients with symptomatic peripheral arterial disease. The low COF group will receive a thin strut-stent with marginal oversizing, whereas the high COF group will receive a thicker strut, stiff-stent with generous oversizing. All employed stent types and sizes are approved for clinical use for the respective anatomical sides, lesions, and indications.

## Methods

### Study design

**Fig. 3:**
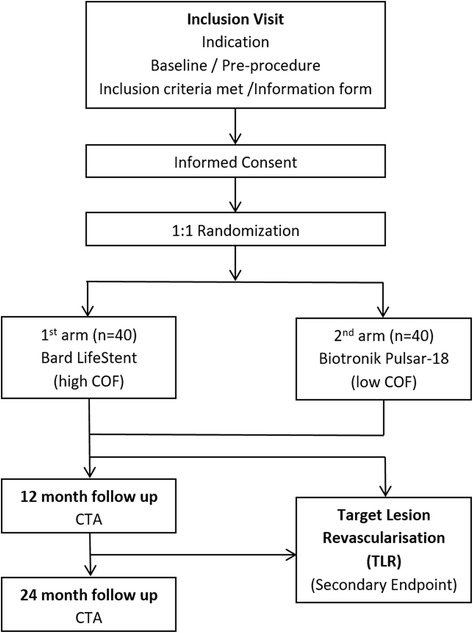
Flow diagram of the study design

The BIOFLEX-COF trial is a monocentric, prospective, quantitative, randomized study which received approval from the Ethics Committee of the Medical University of Vienna (EK Nr. 1026/2015, Ver.5.0, October 2015). The study site is the General Hospital of Vienna of the Medical University of Vienna. The protocol was drafted by the authors of the current manuscript; the sponsor is the Medical University of Vienna, Spitalgasse 23, 1090 Vienna. Patient recruitment started in October 2015. The recruitment period is estimated to be for 24 months, the follow-up period is estimated at 24 months. Figure 3 shows a flow diagram of the study design.

### Stent types

The low COF group receives a thin-strut stent (Pulsar, Biotronik AG, Bülach, Switzerland) with minimal oversizing (according to manufacturer’s instructions), the high COF group receives a stiffer stent (Lifestent Flexstar, Bard Peripheral Vascular Inc., Tempe, AZ, USA) with maximal oversizing (according to manufacturer’s instructions). The stent sizing is based on group allocation and SFA diameter measurements according to a sizing table (Table 1, showing the device sizing according to the vessel diameter). The Pulsar-18 is a laser-cut self-expanding nitinol stent loaded on a low-profile 4 F compatible over-the-wire coaxial delivery system. The stent has a flexible, thin-strut, open-cell design with helically aligned peak-to-valley crowns and six radiopaque markers at each end. It is completely covered with an amorphous silicon carbide coating.

**Table 1:** Stent diameter used according to the vessel diameter

		Nominal stent diameter (mm)		
Vessel diameter (mm)	Pre-dilatation	LifeStent	Pulsar-18	Post-dilatation
4–4.5	3	6	4	4
4.6–5.5	4	6	5	5
5.6–6.5	5	7	6	6
6.6–7.0	6	8	7	7

**Fig. 4:**
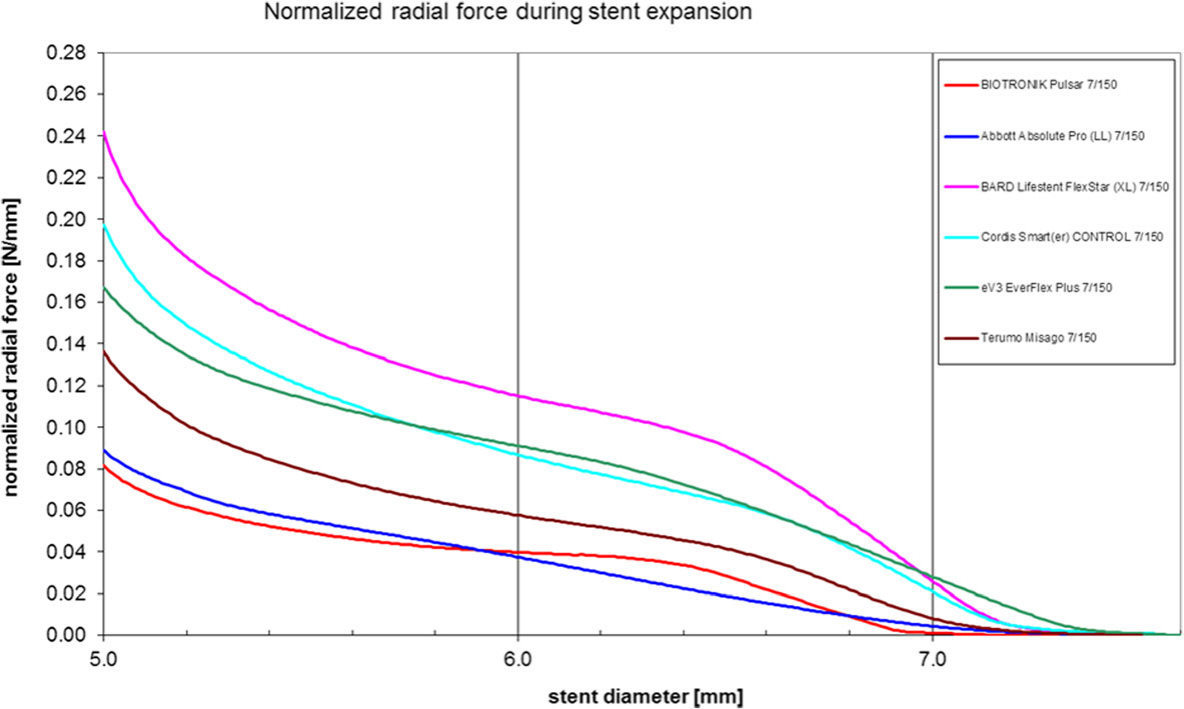
Chronic outward force of different stent fabricates according to stent diameter

The LifeStent Vascular Stent System is comprised of the LifeStent Vascular Stent (stents 20–80 mm) and the LifeStent XL Vascular Stent (stents 100–170 mm). The devices contain self-expanding, flexible, nitinol stents that expand to a preset diameter upon exposure to body temperature. The stents are equivalent to one another in design with only one difference located at the crown section; the LifeStent contain six tantalum radiopaque markers on both the distal and proximal ends of the stent, while the LifeStent XL stent does not have markers. Figure 4 shows the two stent types in detail.

The two stent choices were based on bench test data provided by Biotronik AG on SFA stents from various manufacturers. Using these curves, the COF of each stent can be determined for every diameter below its nominal diameter. The LifeStent shows the steepest COF curve, while the AstronPulsar stent shows the flattest curve. When comparing both stents at a size of 6 × 100 mm the mean values for maximum and minimum RRF are lower for the Pulsar-18 compared to the LifeStent (0.464 and 0.413 N/mm^2^ vs. 1.160 and 1.059 N/mm^2^). Also mean values of maximum and minimum COF are lower for the Pulsar-18 compared to the LifeStent (0.313 and 0.249 N/mm^2^ vs. 0.732 and 0.575 N/mm^2^). The strut dimensions of the Pulsar-18 are 139 × 90 μm compared to 192 × 102 μm for the LifeStent.

### Primary endpoint

Primary endpoint is the amount of in-stent neointima at 1 year, assessed by contrast-enhanced CT angiography (CTA). The volume of neointima inside the stent will be calculated by measuring every 2 mm along the longitudinal axis of the stent: (i) the diameter of the stent in two perpendicular directions and (ii) the diameter of the contrast-enhanced lumen in two perpendicular directions. The difference between these two measurements represents the thickness of the neointima, which will be represented relative to (as percentage of) the stent diameter.

### Secondary objectives

Secondary objectives are the amount of in-stent neointima at 2 years, device- and procedure-related adverse events according to ISO 14155:2011 and target lesion revascularization (TLR) rate. In patients undergoing TLR the amount of in-stent neointima will be assessed by calibrated quantitative angiography at the time of the secondary intervention since no CT angiography will be available for these patients.

Technical success defined as successful stent deployment and absence of significant residual stenosis as well as patent in- and outflow at completion angiography.

### Publication

The investigators plan to publish the trial results in a medical journal and did not enter any agreements restricting publication. Consent form and dataset can be made available on specific request. The study protocol can be found in Additional file 1.

### Enrolment

Eighty subjects who meet all of the inclusion criteria and none of the exclusion criteria with signed patient informed consent will be enrolled. Consent will be obtained by the intervening physician on a dedicated form. The scheduled time for recruitment of all patients is 2 years and is based on recruitment times in multiple previous studies of the center. There will be two follow-up evaluations at 12 and 24 months. The whole study is scheduled to take 4 years. During their participation in the clinical investigation, the patients will be insured against adverse events as defined by legal requirements.

### Inclusion criteria

Patient-related:

1. Subject (or their legal guardian) has read, understood and provided written informed consent, which has been reviewed and approved by the Institutional Review Board.
2. At least 18 years of age.
3. Male, infertile female, or female participants of child-bearing potential practicing an acceptable method of birth control with a negative pregnancy test within 7 days prior to study procedure.
4. Projected life expectancy of greater than 2 years.
5. The ability to comply with protocol follow-up requirements and required testing.

Clinical:

1. Lifestyle-limiting claudication or critical limb ischemia (meeting angiographic entry criteria) affecting a lower extremity (Rutherford stages 2–5). Patients with Rutherford stage 2 are only eligible after unsuccessful conventional and/or medical therapy.
2. Resting ankle-brachial index (ABI) ≤ 0.8 in the study limb.
3. Inflow and/or outflow lesion (including the SFA orifice) - if present - has been treated successfully (treatment in same procedure permissible)

Angiographic and lesion requirements (assessed intraoperatively):

1. TASC A-D lesions with stenoses and/or occlusions ≤ 32 cm (this allows max 2 × 17 cm stents with 1 cm overlap and 0.5 cm overstenting each end of lesion).
2. Popliteal artery is patent 5 cm proximal to the radiographic knee joint line.
3. Reference diameter of 4.0–7.0 mm in proximal and distal treatment segments within the SFA.
4. Patent SFA orifice (the proximal 5 mm after femoral bifurcation).
5. At least one patent (<50% stenotic) inflow vessel present, proven angiographically. Study eligibility is given when inflow lesion has been treated successfully (inflow treatment in same procedure permissible). Successful treatment of inflow lesion is defined as < 50% stenosis without death or severe vascular complication.
6. At least one patent (< 50% stenotic) tibial artery runoff to the ankle present, proven angiographically. Study eligibility is given when runoff vessel lesion has been treated successfully (outflow treatment in same procedure permissible). Successful treatment of outflow lesion is defined as < 50% stenosis without death or severe vascular complication.
7. Guidewire has successfully traversed lesion and is within the true lumen of the distal vessel.

### Exclusion criteria

1. Pregnant or breast-feeding women.
2. Lesion length > 32 cm.
3. Flow-limiting occlusive disease of inflow or outflow arteries that cannot be treated sufficiently.
4. Previous stenting or femoral bypass surgery in the target vessel.
5. Clinical relevant aneurysmal disease of the abdominal aorta, ipsilateral iliac arteries, femoral arteries, or arteries of the knee.
6. Rutherford stage 0, 1, or 6
7. Non-atherosclerotic disease resulting in occlusion (e.g., embolism, Buerger’s disease, vasculitis).
8. Septicemia.
9. Ischemic stroke within the last 3 months.
10. Any previously known coagulation disorder, including hypercoagulability.
11. Morbid obesity or operative scarring that precludes percutaneous approach (at the physician’s discretion).
12. Contraindication to anticoagulation or antiplatelet therapy.
13. Known allergy to medication or contrast media used in this trial, if pre-treatment is not possible (according to the physician’s discretion).
14. Known allergies to stent components, particularly nitinol.
15. Severe calcification of the target lesion that impedes successful pre-dilatation.
16. Current participation in another clinical research trial that has not reached its primary endpoint.
17. The patient is institutionalized based on a legal verdict.

### Pre-interventional evaluation

- Qualifying candidates will undergo the following evaluations:
- Medical history
- Subjective claudication status, walking impairment questionnaire
- Collection of previously available non-invasive studies (CDI ultrasound, CT, MR)
- Blood parameters: platelet count, APTT, PT, creatinine, fibrinogen, hemoglobin, hematocrit, leukocytes, and blood group
- Pregnancy test if indicated.

### Procedure

Vascular access is performed at the investigators discretion under local anesthesia, sedation, or general anesthesia. A contralateral or ipsilateral approach is permissible. A bolus of 5000 units of heparin is administered i.v. Diagnostic angiography is performed from the groin to the ankle (from the aortic bifurcation if no non-invasive imaging is available to document the patency of the inflow). Endovascular treatment of stenosed inflow (including the SFA orifice) and (after successful index lesion transversal) outflow lesions is permissible.

After successful lesion transversal and confirmation of inflow and outflow patency, the patient will be randomized one-to-one by a clinical research assistant (CRA). For this purpose, an online randomization program (www.sealedenvelope.com) will be used to generate a random 1:1 group allocation (block randomization). Only one limb is eligible for inclusion in the study.

A radiopaque calibrated ruler will be present on all radiographic images. Diameter measurements of the proximal and distal landing zone in a un-diseased SFA segment will be performed. These measurements will be used to select the balloon size for pre- and post-dilatation as well as the stent size according to the manufacturers’ stent-sizing table. After pre-dilatation with a subdiameter PTA balloon for 1 minute, the stent will be deployed and the post-dilated. The diameters for the pre- and post-dilatation balloon and the stent diameter will be determined after measuring the diameter of the non-diseased SFA at the proximal end of the intended treatment site. Table 1 gives the respective diameters of the stents for different SFA diameter ranges.

Stents will be deployed beginning with the most distal site. Spot-stenting of multiple lesions is permissible. Any balloon pre-dilated lesion should be covered by the device. Incomplete covered areas, if present, will be evaluated separately.

Stents will be assessed for correct implantation length by observing the relative proximal and distal stent markers with the corresponding delivery system markers. Deviations will be recorded as millimeter elongation or compression.

Completion angiography will be performed from the groin to the ankle (aortic bifurcation to ankle if inflow lesion treatment has been performed). After confirmation of absence of relevant residual stenosis the wire will be removed and radiography of the stent will be performed in two planes differing by ≥ 30 degrees.

Approved closure devices may be utilized at the physician’s discretion. Clopidogrel 75 mg orally once a day will be given for a minimum for 3 months post-procedure. A loading dose of 300 mg will be given immediately after the procedure in patients who did not receive clopidogrel before. Aspirin 100 mg orally once a day will be administered through the study follow-up. In patients with oral anticoagulation aspirin will be the only antiplatelet drug required.

The diameter of implanted stents will be measured at every 2 mm along the stent axis on DICOM images of the respective completion angiography using image processing software (Image J, NIH, MD, USA). To compensate for distortion errors a calibration must be present on each image. By comparing the measured diameter with the nominal diameter of the stent the relative amount of oversizing will be calculated as:

$$ \%\kern0.5em Oversizing\kern0.5em =\kern0.5em \frac{D_n-d}{D_n}\times 100 $$

(Dn = nominal stent diameter, d = actual stent diameter after implantation and post-dilatation)

### Follow-up

At 12 and 24 months, patient receives contrast-enhanced CT angiography, clinical assessment, blood tests, ABI, and BIQ.

### CTA

Patients will be scheduled for two CTA control examinations, after 12 and after 24 months. Stent diameter and true lumen diameter will be measured on DICOM images every 2 mm along the stent axis to quantify the relative amount of in-stent restenosis as:

$$ \%\kern0.5em Restenosis\kern0.5em =\kern0.5em \frac{D_s-{d}_l}{D_s}\times 100 $$

(D_s_ = stent diameter, D_l_ = lumen diameter)

For patients who receive target lesion revascularization or bypass surgery, the pre-operative CTA or angiography will be used to determine the amount of in-stent neointima at the time of the re-intervention.

All metallic stents generate some kind of blooming artifacts at CT imaging with a big variation between the different available stents. To face this issue, several techniques for CT imaging and reconstruction have been proposed, and most of them will be combined in the present study. In this trial, CT angiographies will be obtained with a third-generation Dual Source scanner, and the highest possible spatial resolution will be obtained, since blooming artifacts decrease with increased spatial resolution. Slice collimation will be 2 × 64 × 0.6 mm, and matrix size will be 512 × 512 pixels. Furthermore, image raw data will be reconstructed under application of an iterative reconstruction technique (Advanced Modeled Iterative Reconstruction - ADMIRE) and using a dedicated edge-enhancing kernel. Finally, the three-dimensional reconstruction of multipath-centered curved planar reformations (mCPR) will further facilitate the almost artifact-free assessment of the in-stent lumen.

All measurements will be undertaken on dedicated PACS stations (IMPACS EE R20 XVI SU1, AGFA Healthcare, Mortsel, Belgium) by two independent investigators with a minimum experience of 4 years with the software, blinded to patient and clinical information and group allocation.

**Fig. 5:**
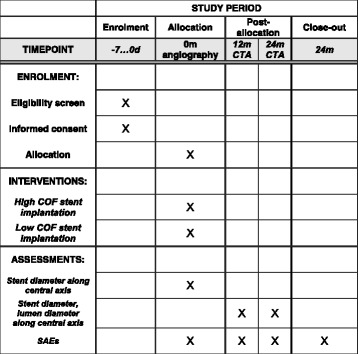
Schedule of enrolment, interventions, and assessments

Figure 5 shows the schedule of enrolment, all interventions and assessments.

### Statistical analysis

Sample size calculation is based on the primary endpoint at 1 and 2 years represented by the percentage of in-stent neointima formation (relative to the stent diameter). Statistical significance between the groups will be tested using a *t* test at a level of significance of 0.05. To detect a difference of 0.8 standard deviations with a statistical power of 90%, a sample size of 34 patients in each group is required. With a maximum dropout rate of 15%, 40 patients will be randomized per group.

A two-sided *t* test will be used to compare amount of neointima between the two treatment groups. In case of non-normally distributed data, appropriate normalizing transformations will be applied. To evaluate the degree of association between the extent of chronic outward force (by using the data presented in Fig. 4 showing COF of stent fabricates according to stent diameter) and the amount of in-stent neointima, a linear regression analysis will be performed independently of the stent allocation group. Primarily, analysis will be based on intention-to-treat. If protocol deviations occur, the analysis will be performed both on an intention-to-treat and on a per protocol basis.

### Trial management and quality assurance

A CRA will perform regular monitoring sessions and interim analyses every 6 months (independent from study investigators and sponsor), in which protocol compliance and correctness of data will be assessed. The CRA will have direct access to medical records, radiographic images, the hospital information system, and all study material for source data verification, will report all serious adverse events (SAEs) to the institutional ethics committee in accordance with all legal and institutional requirements. Case report forms (CRFs) will be generated and stored at the study site. Adherence to the follow-up investigation schedule will be promoted by mailing reminders to the patient 1 month in advance. In addition, the CRA will perform continuous monitoring for adverse events during the study period using the above-mentioned resources and report adverse events and other unintended effects of trial interventions or trial conduct to the institutional ethics committee, which under applicable law has the authority to terminate the trial. Potential relevant protocol modifications must be communicated to the ethics committee and will be communicated to this journal. Confidentiality of personal information will be provided by (a) keeping all documents containing personalized information strictly on site and accessible only by authorized personnel bound by written obligation to confidentiality, (b) anonymizing patient data in the CRFs and the data analysis files. All authors will have unlimited access to the final trial dataset and did not enter agreements that limit such access. Additional file 2 shows the SPIRIT 2013 Checklist as recommended for clinical trial protocols.

## Discussion

The present study is challenging in that it compares two different self-expanding nitinol-stents head-to-head against each other. Very few studies have been successful in this respect, and there are ongoing studies facing this challenge [20], where it remains to be seen whether their sample size calculation can stand up to reality. Consequently, there are currently no prospective studies comparing self-expanding stents with different COF.

The authors believe that in such studies (where a binary endpoint event occurs quite infrequently and in a very similar proportion of patients in either group), binary endpoints do not provide enough power for successful completion without demanding unreasonably high patient numbers. To optimize the power of this study, we dropped both clinical TLR and binary re-stenosis at color-flow Doppler ultrasound (CDUS) as primary endpoints. TLR is a parameter prone to bias, especially if the study team themselves schedule patients for re-intervention, and in claudicants it is further biased on purely subjective estimations by the patient (pain) and by the physician (urgency and resource availability). CDUS is in addition highly operator- and patient-dependent and both parameters are only binary. We selected as outcome parameter the amount of neointima inside the stent accessed by CT angiography. Neointimal volume is calculated based on 20 to 160 individual levels (depending on total stent length every 2 mm along the stent longitudinal access), with each level consisting of four individual measurements (stent diameter in two directions, lumen diameter in two directions). By using the difference between the stent diameter and lumen diameter in a multitude of data points, normal distributions of the results even from skewed data is likely, and the accuracy of the end result as the average of n independent measurements will (theoretically) be improved over the native resolution of a single CT measurement by a factor of √n. Furthermore, in-stent neointima can be detected with CT angiography in virtually every patient after 1 year post implantation, since the detection threshold of the CT is estimated to be at 0.5 mm of luminal narrowing.

A further advantage of CTA assessment of in-stent neointima is the option to locally compare different areas of COF (e.g., in places where some residual stenosis leads to higher compression of the stent and thus to higher chronic outward force, while in other regions where the stent is near its nominal diameter COF might be locally low). The stent diameter (and COF), as well as the amount of neointima, is known every 2 mm along the stent longitudinal access, therefore in-group and in-patient correlations of COF and neointima are possible in post hoc evaluations. If neointima formation is highest at the very same location as high COF, a direct activation of muscle cell proliferation may be assumed. Conversely, if neointima formation occurred downstream from areas with high COF, humoral or paracrine stimulations might be possible.

The study differs further from some similar previous trials in its generous inclusions criteria. Claudicants, patients with tissue loss, as well as diabetic patients are included; inflow and outflow lesion treatment is permissible, TASC A-D lesions are included. This was done in effort to perform the trial on a patient sample that closely represents real-world patients of a specialized endovascular center. The authors believe that it makes little sense to perform trials only on a highly selected collective of participants (representing possibly 1% of all patients) if then (as it is often the case) the conclusions drawn from such selective cohorts are then only generalized to the public anyway.

## Trial status

Recruitment has started in October 2015 and is estimated to be completed in December 2017.

## Additional files

Additional file 1: Patient consent form (German). (PDF 126 kb)

Additional file 2: SPIRIT 2013 checklist. (DOC 123 kb)

## Abbreviations

ABI: Ankle-brachial index
CDUS: Color-flow Doppler ultrasound
COF: Chronic outward force
CRA: Clinical research assistant
CTA: CT angiography
RRF: Radial resistive force
SFA: Superficial femoral artery
TLR: Target lesion revascularization

## References

[1] Lammer J, Zeller T, Hausegger KA, Schaefer PJ, Gschwendtner M, Mueller-Huelsbeck S, Rand T, Funovics M, Wolf F, Rastan A, Gschwandtner M, Puchner S, Beschorner U, Ristl R, Schoder M (2015). Sustained benefit at 2 years for covered stents versus bare-metal stents in long SFA lesions: the VIASTAR trial. Cardiovasc Intervent Radiol.

[2] Jaff MR, White CJ, Hiatt WR, Fowkes GR, Dormandy J, Razavi M, Reekers J, Norgren L (2015). An update on methods for revascularization and expansion of the tasc lesion classification to include below-the-knee arteries: a supplement to the inter-society consensus for the management of peripheral arterial disease (tasc ii): the TASC Steering Committee(.). Ann Vasc Dis.

[3] Saxon RR, Dake MD, Volgelzang RL, Katzen BT, Becker GJ (2008). Randomized, multicenter study comparing expanded polytetrafluoroethylene-covered endoprosthesis placement with percutaneous transluminal angioplasty in the treatment of superficial femoral artery occlusive disease. J Vasc Interv Radiol.

[4] Rastan A, Krankenberg H, Baumgartner I, Blessing E, Muller-Hulsbeck S, Pilger E, Scheinert D, Lammer J, Beschorner U, Noory E, Neumann FJ, Zeller T (2015). Stent placement vs. Balloon angioplasty for popliteal artery treatment: two-year results of a prospective, multicenter, randomized trial. J Endovasc Ther.

[5] Dick P, Wallner H, Sabeti S, Loewe C, Mlekusch W, Lammer J, Koppensteiner R, Minar E, Schillinger M (2009). Balloon angioplasty versus stenting with nitinol stents in intermediate length superficial femoral artery lesions. Catheter Cardiovasc Interv.

[6] Krankenberg H, Schluter M, Steinkamp HJ, Burgelin K, Scheinert D, Schulte KL, Minar E, Peeters P, Bosiers M, Tepe G, Reimers B, Mahler F, Tubler T, Zeller T (2007). Nitinol stent implantation versus percutaneous transluminal angioplasty in superficial femoral artery lesions up to 10 cm in length: the femoral artery stenting trial (fast). Circulation.

[7] Dake MD, Ansel GM, Jaff MR, Ohki T, Saxon RR, Smouse HB, Zeller T, Roubin GS, Burket MW, Khatib Y, Snyder SA, Ragheb AO, White JK, Machan LS, Zilver PTXI (2011). Paclitaxel-eluting stents show superiority to balloon angioplasty and bare metal stents in femoropopliteal disease: twelve-month zilver ptx randomized study results. Circ Cardiovasc Interv.

[8] Deloose K, Lauwers K, Callaert J, Maene L, Keirse K, Verbist J, Peeters P, Bosiers M (2013). Drug-eluting technologies in femoral artery lesions. J Cardiovasc Surg (Torino).

[9] Brodmann M (2014). Prime time for drug eluting balloons in SFA interventions?. J Cardiovasc Surg (Torino).

[10] Minar E, Schillinger M (2012). Innovative technologies for sfa occlusions: drug coated balloons in sfa lesions. J Cardiovasc Surg (Torino).

[11] Schmidt W, Wissgott C, Andresen R, Behrens P, Schmitz KP (2011). Performance characteristics of modern self-expanding nitinol stents indicated for SFA. Röfo.

[12] Johnston CR, Lee K, Flewitt J, Moore R, Dobson GM, Thornton GM (2010). The mechanical properties of endovascular stents: an in vitro assessment. Cardiovasc Eng.

[13] Cho H, Nango M, Sakai Y, Sohgawa E, Kageyama K, Hamamoto S, Kitayama T, Yamamoto A, Miki Y (2014). Neointimal hyperplasia after stent placement across size-discrepant vessels in an animal study. Jpn J Radiol.

[14] Zhao HQ, Nikanorov A, Virmani R, Jones R, Pacheco E, Schwartz LB (2009). Late stent expansion and neointimal proliferation of oversized nitinol stents in peripheral arteries. Cardiovasc Intervent Radiol.

[15] Freeman JW, Snowhill PB, Nosher JL (2010). A link between stent radial forces and vascular wall remodeling: The discovery of an optimal stent radial force for minimal vessel restenosis. Connect Tissue Res.

[16] Saguner AM, Traupe T, Raber L, Hess N, Banz Y, Saguner AR, Diehm N, Hess OM (2012). Oversizing and restenosis with self-expanding stents in iliofemoral arteries. Cardiovasc Intervent Radiol.

[17] Kirsch EC, Khangure MS, Morling P, York TJ, McAuliffe W (2002). Oversizing of self-expanding stents: Influence on the development of neointimal hyperplasia of the carotid artery in a canine model. AJNR Am J Neuroradiol.

[18] Vorwerk D, Redha F, Neuerburg J, Clerc C, Gunther RW (1994). Neointima formation following arterial placement of self-expanding stents of different radial force: experimental results. Cardiovasc Intervent Radiol.

[19] Saxon RR, Chervu A, Jones PA, Bajwa TK, Gable DR, Soukas PA, Begg RJ, Adams JG, Ansel GM, Schneider DB, Eichler CM, Rush MJ (2013). Heparin-bonded, expanded polytetrafluoroethylene-lined stent graft in the treatment of femoropopliteal artery disease: 1-year results of the VIPER (Viabahn Endoprosthesis with Heparin Bioactive Surface in the Treatment of Superficial Femoral Artery Obstructive Disease) trial. J Vasc Interv Radiol.

[20] Goueffic Y, Kaladji A, Guyomarch B, Montagne C, Fairier D, Gestin S, Riche VP, Vent PA, Chaillou P, Costargent A, Patra P (2014). Bare metal stent versus paclitaxel eluting stent for intermediate length femoropopliteal arterial lesions (battle trial): Study protocol for a randomized controlled trial. Trials.

