# Supplementary material for: Self-expanding nitinol stents of high versus low chronic outward force in de novo femoropopliteal occlusive arterial lesions (BIOFLEX-COF trial): study protocol for a randomized controlled trial
Source: Trials. 2017 Dec 14;18:594. doi: 10.1186/s13063-017-2338-0 (PMC5729260; doi:10.1186/s13063-017-2338-0)
Supplement: Supplementary file 1 — Patient consent form (German). (PDF 126 kb) [file 13063_2017_2338_MOESM1_ESM.pdf]

PatientInneninformation[1] und Einwilligungserklärung zur Teilnahme an der klinischen Prüfung

Prospektive[2], randomisierte[3], einfach verblindete[4] Studie zur Untersuchung des Einflusses der Stent-Radialkraft[5] auf die In-Stent Restenoserate in der Arteria femoralis superficialis[6] – COPIST (Chronic Outward Force and Patency In Stents) Trial

Sehr geehrte Patientin, sehr geehrter Patient!

Wir laden Sie ein an der oben genannten klinischen Prüfung teilzunehmen. Die Aufklärung darüber erfolgt in einem ausführlichen ärztlichen Gespräch.

Ihre Teilnahme an dieser klinischen Prüfung erfolgt freiwillig. Sie können jederzeit ohne Angabe von Gründen aus der Studie ausscheiden. Die Ablehnung der Teilnahme oder ein vorzeitiges Ausscheiden aus dieser Studie hat keine nachteiligen Folgen für Ihre medizinische Betreuung.

Klinische Prüfungen sind notwendig, um verlässliche neue medizinische Forschungsergebnisse zu gewinnen. Unverzichtbare Voraussetzung für die Durchführung einer klinischen Prüfung ist jedoch, dass Sie Ihr Einverständnis zur Teilnahme an dieser klinischen Prüfung schriftlich erklären. Bitte lesen Sie den folgenden Text als Ergänzung zum Informationsgespräch mit Ihrem Prüfarzt sorgfältig durch und zögern Sie nicht Fragen zu stellen.

Bitte unterschreiben Sie die Einwilligungserklärung nur

- wenn Sie Art und Ablauf der klinischen Prüfung vollständig verstanden haben,
- wenn Sie bereit sind, der Teilnahme zuzustimmen und
- wenn Sie sich über Ihre Rechte als Teilnehmer an dieser klinischen Prüfung im Klaren sind.

---

[1] Wegen der besseren Lesbarkeit wird im weiteren Text zum Teil auf die gleichzeitige Verwendung weiblicher und männlicher Personenbegriffe verzichtet. Gemeint und angesprochen sind – sofern zutreffend – immer beide Geschlechter.

[2] Prospektive Studie: Überprüfung der unbewiesenen Annahme der Wirksamkeit einer Behandlungsmethode unter vorheriger Festlegung, welche Annahme geprüft werden soll.

[3] Randomisieren: Zufällige Verteilung in verschiedene Behandlungsgruppen.

[4] Einfach verblindet: Sie wissen nicht, welcher Behandlungsgruppe Sie zugeteilt werden, Ihr Arzt ist darüber informiert.

[5] Kraft mit der sich ein Stent ausdehnt und die er von innen auf die Gefäßwand ausübt

[6] Wiederverschluss der Blutgefäße im Oberschenkel im behandelten Abschnitt.

Zu dieser klinischen Prüfung, sowie zur Patienteninformation und Einwilligungserklärung wurde von der zuständigen Ethikkommission eine befürwortende Stellungnahme abgegeben.

1. Was ist der Zweck der klinischen Prüfung?

Die Symptome, unter denen Sie leiden, resultieren aus einer Verengung oder einem Verschluss Ihrer Blutgefäße, die zu einer Beeinträchtigung des Blutflusses zu Ihrem Bein führt. Um den Blutfluss wieder zu verbessern und die Symptome zu lindern, ist eine Öffnung der Blutgefäße erforderlich. Dies kann durch das Einsetzten eines Stents in das Blutgefäß erreicht werden. Ein Stent ist ein kleines Metallgerüst, das ausgeweitet wird und dauerhaft gegen die Gefäßwand gepresst wird, um das Gefäß offen zu halten. Diese Art der Behandlung wird routinemäßig bei Ihrer Erkrankung durchgeführt und wird Ihnen von Ihrem Arzt detailliert erklärt.

Sie können sich einen Stent vorstellen wie eine Feder, die von innen Ihr Blutgefäß aufdehnt. Die Kraft mit der ein Stent sich ausdehnt, im weiteren Text als Radialkraft bezeichnet, ist unterschiedlich zwischen verschiedenen Stentfabrikaten. Außerdem spielt der Durchmesser des Stents eine wichtige Rolle. Ein Stent mit großem Durchmesser in einem Blutgefäß mit kleinem Durchmesser übt eine stärkere Kraft auf die Gefäßwand aus als einer mit kleinerem Durchmesser.

Der Zweck dieser klinischen Prüfung ist den Einfluss der Radialkraft auf die Wiederverschlussrate (die Häufigkeit, mit der trotz der Behandlung mit einem Stent, erneut dieselben Beschwerden in ihrem Bein auftreten) von Stents in der Oberschenkelarterie zu untersuchen. Zu diesem Zweck wird ein Stent mit hoher Radialkraft (Bard® LifeStent) mit einem mit geringer Radialkraft (Biotronik® Pulsar-18) verglichen. Beide Stents werden routinemäßig bei Patienten mit Ihren Beschwerden verwendet, allerdings ist momentan nicht klar ob es besser ist Stents mit hoher oder mit geringer Radialkraft zu verwenden. Zur Klärung dieser Frage soll diese Studie einen Beitrag leisten. Beide Stents werden entsprechend eines Routineeingriffes, wie in der Benutzeranleitung empfohlen, verwendet.

2. Welche anderen Behandlungsmöglichkeiten gibt es?

Zur Behandlung Ihrer Erkrankung stehen **stattdessen auch** die folgenden Möglichkeiten zur Verfügung:

- Sie könnten mit einem Stent oder Ballonkatheter behandelt werden, ohne an einer Studie teilzunehmen.
- Sie könnten durch eine rein medizinische Therapie behandelt werden (Medikamente, Änderung Ihres Lebensstils zur Verringerung Ihres Blutdrucks, Gewichtsreduzierung usw.).
- Sie könnten durch einen chirurgischen Eingriff behandelt werden.

3. Wie läuft die klinische Prüfung ab?

Diese klinische Prüfung wird an unserer Klinik durchgeführt, und es werden insgesamt ungefähr 80 Personen daran teilnehmen.

Vor Aufnahme in diese klinische Prüfung wird die Vorgeschichte Ihrer Krankheit erhoben, und Sie werden einer umfassenden ärztlichen Untersuchung unterzogen.

Ihre Teilnahme an dieser klinischen Prüfung wird voraussichtlich 2 Jahre dauern.

Im Rahmen dieser klinischen Prüfung werden zwei bereits zugelassene Stents miteinander verglichen: der Pulsar-18 der Firma Biotronik AG, 8180 Bülach, Schweiz und der Bard® LifeStent der Firma Angiomed GmbH & Co. Medizintechnik KG, 76227 Karlsruhe, Deutschland. Im Falle Ihrer Teilnahme werden Sie entweder mit dem Biotronik® Pulsar-18 oder dem Bard® LifeStent behandelt. Welches der Produkte im Falle Ihrer Teilnahme angewendet wird, entscheidet ein zuvor festgelegtes Zufallsverfahren, vergleichbar mit dem Werfen einer Münze; dieses Verfahren wird Randomisieren genannt. Die jeweilige Wahrscheinlichkeit einen der beiden Stents zu erhalten beträgt 50%. Sie wissen nicht, welcher Stent zur Behandlung verwendet wurde. Sollte es aber notwendig werden, kann Ihr Prüfarzt Ihnen jederzeit mitteilen, mit welchem der beiden Stent Sie behandelt wurden.

Eine Reihe von Untersuchungen und Eingriffen werden im Zuge Ihrer Behandlung durchgeführt, gleichgültig, ob Sie nun an dieser klinischen Prüfung teilnehmen oder nicht. Diese werden von Ihrem Prüfarzt im Rahmen des üblichen ärztlichen Aufklärungsgespräches mit Ihnen besprochen. Es werden folgende Untersuchungen routinemäßig durchgeführt, die nachstehend genauer erklärt werden:

<u>Nach 12 und 24 Monaten</u>

• Duplex-Ultraschalluntersuchung des behandelten Beines

• Klinische Untersuchung

• Blutabnahme zur Bestimmung einiger Blutwerte

• Bestimmung der schmerzfreien Gehleistung: Unter dieser gleichförmigen Belastung misst man die Strecke bis zum Beginn der Schmerzen (schmerzfreie Gehstrecke) und die Strecke bis zur Gehunfähigkeit wegen Schmerzen (absolute Gehstrecke) in Metern.

• Computertomographie (CT) der Beinarterien

• Knöchel-Arm-Index-Messung im Ruhezustand für das behandelte Bein: Der Knöchel-Arm-Index wird zur Abschätzung des Blutflusses im behandelten Bein bestimmt. Dafür wird Ihr Blutdruck am Oberarm und am Knöchel ermittelt.

Wenn Sie sich zur Teilnahme an dieser Studie entschließen, müssen Sie nach 12 und 24 Monaten zur Nachuntersuchung in die Klinik kommen. Auch bei den Nachuntersuchungen in der Klinik wird Sie der behandelnde Arzt zu Ihrem Befinden seit der Durchführung des Eingriffs befragen.

Es ist sehr wichtig, dass Sie zu den Nachkontrollen kommen. Ihr Arzt kann dann eventuelle Nebenwirkungen der Behandlung erkennen. Außerdem kann nur durch die Nachkontrollen die Wirksamkeit der Behandlung beurteilt werden.

Die Gesamtdauer Ihrer Studienteilnahme beträgt zwei Jahre. Nach Ende der Studie werden Sie in Absprache mit Ihrem behandelnden Arztes gemäß dem üblichen medizinischen Standard weiter betreut.

Alle oben genannten Untersuchungen sind Routine-Nachkontrollen und werden auch ohne Teilnahme an dieser Studie bei Ihnen durchgeführt. Jährliche Nachkontrollen sind nach einer Stentimplantation in jedem Fall vorgesehen. Keine der oben genannten Untersuchungen wird rein zum Zwecke der Studie durchgeführt.

4. Was ist der Pulsar-18 der Firma Biotronik bzw. der LifeStent der Firma Angiomed?

Sowohl der Pulsar-18 der Firma Biotronik als auch der LifeStent der Firma Angiomed sind Medizinprodukte, die bereits zugelassen sind. Beide Medizinprodukte werden gegenwärtig bei der Behandlung von Verschlüssen der Blutgefäße im Bein angewendet und werden im klinischen Routinebetrieb verwendet.

5. Worin liegt der Nutzen einer Teilnahme an der Klinischen Prüfung?

Da die Behandlung die Sie im Rahmen der Studie erhalten eine Routinebehandlung darstellt ist durch die Teilnahme an der Studie kein unmittelbarer Nutzen für Sie zu erwarten.

6. Gibt es Risiken, Beschwerden und Begleiterscheinungen?

Ihr Arzt hat mit Ihnen über die Risiken der Behandlung zur Dehnung der Beingefäße mit einem Stent (Stentdilatation) gesprochen. Da beide Stents bereits zugelassene Medizinprodukte sind und sie im Rahmen dieser Studie beide innerhalb ihrer Zulassung angewendet werden, sind die Risiken bei Teilnahme an der Studie prinzipiell nicht höher als bei einer vergleichbaren Behandlung außerhalb der Studie.

Wie oben erwähnt, gehört die Stentdilatation zu den Standardbehandlungsverfahren bei erkrankten Beinblutgefäßen. Allerdings kann es jederzeit zu Komplikationen kommen. Über die Stentdilatation und deren Risiken werden Sie von Ihrem behandelnden Arzt bei der allgemeinen Aufklärung über den Eingriff informiert und sind im Folgenden näher beschrieben. Die potenziellen Risiken, die mit der Behandlung zur Erweiterung der Beingefäße verbunden sind und sehr selten (weniger als 1 Behandelter von 10.000) auftreten, sind: Wundheilungsstörungen oder Wundinfektionen. Die folgenden Nebenwirkungen können selten (1 bis 10 Behandelte von 10.000) vorkommen: Herzrhythmusstörungen, allergische Reaktion auf Medikamente, die während des Verfahrens verabreicht werden, Tod, Fieber, Nieren-Komplikationen, erneute Verengung des behandelten Gefäßes, Krampfanfall. Die folgenden Nebenwirkungen können gelegentlich (1 bis 10 Behandelte von 1.000) auftreten: Amputation des behandelten Beines, Blutungen, Blutdruckschwankungen, Blut / Kreislauf-Komplikationen, Herzbeschwerden, Schmerzen in den Beinen, Übelkeit, Schlaganfall, Komplikationen am behandelten Gefäß. Häufig (1 bis 10 Behandelte von 100) kann es zu Unwohlsein während des Eingriffs kommen.

Während des Eingriffs wird Ihr Arzt eine Flüssigkeit verwenden, die die Sichtbarkeit der Blutgefäße in dem zu behandelnden Bereich verbessern wird. Diese Flüssigkeit, genannt Kontrastmittel, kann bei Patienten mit vorbestehender Nierenerkrankung zu Problemen mit der Nierenfunktion führen. Des

Weiteren kann Kontrastmittel den Blutdruck erhöhen oder senken, zu Herzrhythmusstörungen führen, allergische Reaktionen auslösen, oder zu Übelkeit und Erbrechen führen. Bei der Behandlung mit einem Stent ist die Verwendung von Kontrastmittel in jedem Fall notwendig, egal ob Sie sich dazu entschließen an der Studie teilzunehmen, oder nicht.

7. Zusätzliche Einnahme von Arzneimitteln?

Medikamente oder Medizinprodukte, von denen der Prüfarzt noch nichts weiß, dürfen Sie – außer bei Notfällen – nur nach Rücksprache mit Ihrem Prüfarzt einnehmen oder verwenden. Wenn Sie von anderen Ärzten behandelt werden, müssen Sie diese über Ihre Teilnahme an der klinischen Prüfung informieren. Auch Ihr Prüfarzt muss über jede medizinische Behandlung, die Sie durch einen anderen Arzt während der klinischen Prüfung erhalten, informiert werden.

8. Was ist zu tun beim Auftreten von Symptomen, Begleiterscheinungen und/oder Verletzungen?

Sollten im Verlauf der klinischen Prüfung irgendwelche Symptome, Begleiterscheinungen oder Verletzungen auftreten, müssen Sie diese Ihrem Prüfarzt mitteilen, bei schwerwiegenden Begleiterscheinungen umgehend, ggf. telefonisch (Telefonnummern, etc. siehe unten).

9. Versicherung

Als Teilnehmer an dieser klinischen Prüfung besteht für Sie der gesetzlich vorgeschriebene verschuldensunabhängige Versicherungsschutz (Personenschadenversicherung gemäß § 47 Medizinproduktegesetz, der alle Schäden abdeckt, die an Ihrem Leben oder Ihrer Gesundheit durch die an Ihnen durchgeführten Maßnahmen der klinischen Prüfung verursacht werden können, mit Ausnahme von Schäden auf Grund von Veränderungen des Erbmaterials in Zellen der Keimbahn.

Die Versicherung wurde für Sie bei der Zürich Versicherungs-Aktiengesellschaft, Schwarzenbergplatz 15, 1010 Wien, Tel. +43 1 50125-0 unter der Polizzennummer 07229622-2 abgeschlossen. Auf Wunsch können Sie in die Versicherungsunterlagen Einsicht nehmen.

Im Schadensfall können Sie sich direkt an den Versicherer wenden und Ihre Ansprüche selbständig geltend machen. Für den Versicherungsvertrag ist österreichisches Recht anwendbar, die Versicherungsansprüche sind in Österreich einklagbar.

Zur Unterstützung können Sie sich auch an die Patientenanwaltschaft oder Patienten Vertretung wenden: Tel.: + 43 1 587 12 04; Adresse: WPPA, Schönbrunner Straße 108; 1050 Wien; Österreich

Um den Versicherungsschutz nicht zu gefährden

- dürfen Sie sich während der Dauer der klinischen Prüfung einer anderen medizinischen Behandlung nur im Einvernehmen mit Ihrem behandelnden Prüfarzt unterziehen **(ausgenommen davon sind Notfälle).** Dies gilt auch für die zusätzliche Einnahme von Medikamenten oder die Teilnahme an einer anderen Studie.

- müssen Sie dem behandelnden Prüfarzt - oder der oben genannten Versicherungsgesellschaft - eine Gesundheitsschädigung, die als Folge der klinischen Prüfung eingetreten sein könnte, unverzüglich mitteilen.
- müssen Sie alles Zumutbare tun um Ursache, Hergang und Folgen des Versicherungsfalles aufzuklären und den entstandenen Schaden gering zu halten. Dazu gehört ggf. auch, dass Sie Ihre behandelnden Ärzte ermächtigen, vom Versicherer geforderte Auskünfte zu erteilen.

10. Informationen für gebärfähige Frauen – Schwangerschaftstest

Schwangere und stillende Frauen dürfen an dieser klinischen Prüfung NICHT teilnehmen.

11. Wann wird die klinische Prüfung vorzeitig beendet?

Sie können jederzeit auch ohne Angabe von Gründen, Ihre Teilnahmebereitschaft widerrufen und aus der klinischen Prüfung ausscheiden ohne dass Ihnen dadurch irgendwelche Nachteile für Ihre weitere medizinische Betreuung entstehen.

Ihr Prüfarzt wird Sie über alle neuen Erkenntnisse, die in Bezug auf diese klinische Prüfung bekannt werden, und für Sie wesentlich werden könnten, umgehend informieren. Auf dieser Basis können Sie dann Ihre Entscheidung zur **weiteren** Teilnahme an dieser klinischen Prüfung neu überdenken.

Es ist aber auch möglich, dass Ihr Prüfarzt (oder gegebenenfalls der Auftraggeber dieser klinischen Prüfung) entscheidet, Ihre Teilnahme an der klinischen Prüfung vorzeitig zu beenden, ohne vorher Ihr Einverständnis einzuholen. Die Gründe hierfür können sein:

a) Sie können den Erfordernissen der Klinischen Prüfung nicht entsprechen;

b) Ihr Prüfarzt hat den Eindruck, dass eine weitere Teilnahme an der klinischen Prüfung nicht in Ihrem Interesse ist;

Sofern Sie sich dazu entschließen, vorzeitig aus der klinischen Prüfung auszuscheiden, oder Ihre Teilnahme aus einem der oben genannten Gründe vorzeitig beendet wird, ist es für Ihre eigene Sicherheit wichtig, dass Sie sich einer normalen Kontrolluntersuchung unterziehen. Diese besteht meistens aus einer körperlichen Untersuchung sowie aus Laboruntersuchungen und einer Duplex-Ultraschalluntersuchung.

12. In welcher Weise werden die im Rahmen dieser klinischen Prüfung gesammelten Daten verwendet?

Sofern gesetzlich nicht etwas anderes vorgesehen ist, haben nur die Prüfärzte und deren Mitarbeiter Zugang zu den vertraulichen Daten, in denen Sie namentlich genannt werden („personenbezogene“ Daten). Weiters können Beauftragte von in- und ausländischen Gesundheitsbehörden, der zuständigen Ethikkommission, sowie – wenn zutreffend – des Auftraggebers der klinischen Prüfung Einsicht in diese Daten nehmen, um die Richtigkeit der Aufzeichnungen zu überprüfen. Diese Personen unterliegen einer gesetzlichen Verschwiegenheitspflicht.

Die Weitergabe der Daten im In- und Ausland erfolgt ausschließlich zu statistischen Zwecken in verschlüsselter (nur „indirekt personenbezogener“) oder nicht personenbezogener („anonymisierter“) Form, das heißt, Sie werden nicht namentlich genannt. Auch in etwaigen Veröffentlichungen der Daten dieser klinischen Prüfung werden Sie nicht namentlich genannt.

Die Prüfärzte und ihre Mitarbeiter unterliegen im Umgang mit den Daten den Bestimmungen des österreichischen Datenschutzgesetzes 2000 in der jeweils geltenden Fassung.

Wenn Sie Ihre Einwilligung zurückziehen und damit Ihre Teilnahme vorzeitig beenden, werden keine neuen Daten mehr über Sie erhoben. Auf Grund gesetzlicher Dokumentationspflichten (Arzneimittel- bzw. Medizinproduktegesetz) kann jedoch weiterhin für einen gesetzlich festgelegten Zeitraum eine Einsichtnahme in Ihre personenbezogenen Daten zu Prüfzwecken durch autorisierte, zur Verschwiegenheit verpflichtete Personen erfolgen.

13. Entstehen für die Teilnehmer Kosten? Gibt es einen Kostenersatz oder eine Vergütung?

Durch Ihre Teilnahme an dieser klinischen Prüfung entstehen für Sie keine zusätzlichen Kosten.

14. Möglichkeit zur Diskussion weiterer Fragen

Für weitere Fragen im Zusammenhang mit dieser klinischen Prüfung stehen Ihnen Ihr Prüfarzt und seine Mitarbeiter gerne zur Verfügung. Auch Fragen, die Ihre Rechte als Patient und Teilnehmer an dieser klinischen Prüfung betreffen, werden Ihnen gerne beantwortet.

Name der Kontaktperson: Ass.-Prof. Doz. Dr. Martin Funovics

Erreichbar unter: Tel. +43 1 40 400 58020

Name der Kontaktperson: Dr. Alexander Wressnegger

Erreichbar unter: Tel. +43 1 40 400 57970

15. Einwilligungserklärung

Name des Patienten in Druckbuchstaben: ....

Geb.Datum: .... Code: ....

Ich erkläre mich bereit, an der klinischen Prüfung Prospektive, randomisierte, einfach verblindete Studie zur Untersuchung des Einflusses der Stent-Radialkraft auf die In-Stent Restenoserate in der Arteria femoralis superficialis – COPIST (Chronic Outward Force and Patency In Stents) Trial teilzunehmen.

Ich bin in einem persönlichen Gespräch durch den Prüfarzt

....
Name der Ärztin / des Arztes

ausführlich und verständlich über mögliche Belastungen und Risiken, sowie über Wesen, Bedeutung und Tragweite der klinischen Prüfung, die bestehende Versicherung sowie die sich für mich daraus ergebenden Anforderungen aufgeklärt worden. Ich habe darüber hinaus den Text dieser Patientenaufklärung und Einwilligungserklärung, die insgesamt 9 Seiten umfasst gelesen. Aufgetretene Fragen wurden mir vom Prüfarzt verständlich und genügend beantwortet. Ich hatte ausreichend Zeit, mich zu entscheiden. Ich habe zurzeit keine weiteren Fragen mehr.

Ich werde den ärztlichen Anordnungen, die für die Durchführung der klinischen Prüfung erforderlich sind, Folge leisten, behalte mir jedoch das Recht vor, meine freiwillige Mitwirkung jederzeit zu beenden, ohne dass mir daraus Nachteile für meine weitere medizinische Betreuung entstehen.

Ich bin zugleich damit einverstanden, dass meine im Rahmen dieser klinischen Prüfung ermittelten Daten gespeichert werden. Mir ist bekannt, dass zur Überprüfung der Richtigkeit der Datenaufzeichnung Beauftragte der zuständigen Behörden, der Ethikkommission und ggf. des Auftraggebers beim Prüfarzt Einblick in meine personenbezogenen Krankheitsdaten nehmen dürfen.

Sollte ich meine Teilnahme an dieser Studie widerrufen oder wird meine Teilnahme an der Studie durch den Sponsor oder den Prüfarzt vorzeitig beendet, so willige ich ein, dass die bis zu diesem Zeitpunkt erhobenen Daten weiterhin verwendet werden dürfen, soweit dies erforderlich ist, um

a) sicherzustellen, dass meine schutzwürdigen Interessen nicht beeinträchtigt werden

und – wenn zutreffend –

b) der gesetzlichen Pflicht zur Vorlage vollständiger Zulassungsunterlagen und den gesetzlichen Dokumentationspflichten zu entsprechen.

Beim Umgang mit den Daten werden die Bestimmungen des Datenschutzgesetzes 2000 beachtet.

Für den Fall, dass ich aus der Studie ausscheide, bin ich einverstanden, dass meine Proben weiterhin aufbewahrt und analysiert werden, wie in dieser Information und – wenn zutreffend – in den Informationen zu den Substudien beschrieben:

☐ ja ☐ nein

Eine Kopie dieser Patienteninformation und Einwilligungserklärung habe ich erhalten. Das Original verbleibt beim Prüfarzt.

....
(Datum und Unterschrift des Patienten)

....
(Datum, Name und Unterschrift des verantwortlichen Prüfarztes)

(Der Patient erhält eine unterschriebene Kopie der Patienteninformation und Einwilligungserklärung, das Original verbleibt im Studienordner des Prüfarztes.)
